# Cholera due to exposure in Europe associated with consumption of holy water from Ethiopia, January to February 2025

**DOI:** 10.2807/1560-7917.ES.2025.30.14.2500234

**Published:** 2025-04-10

**Authors:** Christina Frank, Claire Jenkins, Jana-Marie Weis, Anja Brilmayer, Anja Schoeps, Susann Dupke, Hendrik Wilking, Parisha Katwa, Satheesh Nair, Clare Barker, Derren Ready, Gauri Godbole, Susan Hopkins, Hilary Kirkbride

**Affiliations:** 1 Department for Infectious Disease Epidemiology, Robert Koch Institute, Berlin, Germany; 2 United Kingdom (UK) Health Security Agency, London, United Kingdom; 3 Bad Kreuznach Public Health Office, Bad Kreuznach, Germany; 4 Federal State Agency for Consumer and Health Protection Rhineland-Palatinate, Koblenz, Germany; 5 Centre for Biological Threats and Special Pathogens, Robert Koch Institute, Berlin, Germany

**Keywords:** Cholera, *Vibrio cholerae* O1, travel, Africa, holy water, autochthonous infection, antimicrobial resistance

## Abstract

In February 2025, public health agencies in Germany and the United Kingdom (UK) reported four cases of domestically acquired cholera caused by consumption of holy water imported from Ethiopia, and a further three cases in travellers to Ethiopia. Multidrug-resistant *Vibrio cholerae* O1, linked to recent outbreaks in Eastern and Middle Africa, was detected in clinical specimens and the holy water. In cholera-endemic regions, visitors should drink potable water and should not bring bottled water back from their travels.

In February 2025, the Robert Koch Institute (RKI) in Berlin and the UK Health Security Agency (UKHSA) in London identified seven patients infected with the same multidrug-resistant (MDR) strain of *Vibrio cholerae* serogroup O1. At least four of the seven cases became infected in Europe. We aimed to review the epidemiology, identify the source and most likely route of transmission and provide recommendations to prevent further infections.

## Cholera outbreak in Ethiopia

The current cholera outbreak in Ethiopia started in August 2022, and by 9 February 2025, a total of 58,381 cases and 726 deaths had been reported [1]. On 6 February 2025, a resurgence of the cholera outbreak was reported in the Amhara region with 163 cases and three deaths [2]. One contamination source was identified at the Bermel Giorgis (also spelled ‘Georgis’) holy well, in the Quara district [3]. The holy well is a site of pilgrimage attracting visitors from across the globe where rituals such as cleansing with holy water ‘tsebel’ are undertaken. The holy water is consumed or used for bathing for physical or spiritual healing. Although it is not sold commercially, it can be taken home by pilgrims.

## Description of the European patients linked to the Ethiopian outbreak

In Germany, the suspicion of cholera was raised and communicated on 25 February 2025 via EpiPulse, the European surveillance portal for infectious diseases (https://www.ecdc.europa.eu/en/publications-data/epipulse-european-surveillance-portal-infectious-diseases) for three patients, in the age group 40–65 years, all identified as having Ethiopian ethnicity, with exposure to water from the holy well in Bermel Giorgis. Two of the patients had travelled in Ethiopia during 4 weeks in January 2025 where they acquired a small plastic bottle of water from the holy well in Bermel Giorgis. On returning to Germany, they consumed the water together with a third person, who had not travelled. The two travellers consumed the water, while the third person received splashes of water in the face including the lips and may have ingested some water. In early February 2025, all three individuals simultaneously developed acute watery diarrhoea and were hospitalised with vomiting and profuse watery stools. One patient required intensive care, but all recovered. Toxigenic *V. cholerae* O1, biovar El Tor was isolated from faecal specimens from the three patients and from the residual holy water consumed by the German cases. All three infections were considered autochthonous.

The UKHSA identified four patients, resident in the UK (aged in their 20s to 60s) infected with toxigenic *V. cholerae* O1 biovar El Tor isolated from stool samples. Three patients independently reported recent travel to Ethiopia, two reporting travel to the Amhara region, with one of these specifically reporting a 9-day religious trip to the holy well in Bermel Georgis during which local water was used for food preparation and washing. A fourth UK patient did not travel outside of the UK but reported that they drank holy water from Ethiopia, brought back to the UK by the third UK patient, who also became ill after consuming the water in the UK. The travellers returned to the UK from Ethiopia between the third tercile of January and mid-February 2025, and symptom onset for all patients fell within that timeframe. Three were admitted to hospital and reported symptoms of watery diarrhoea, vomiting and dehydration, one needed intensive care for fluid resuscitation. One case had a *Campylobacter* co-infection, and another had a co-infection with malaria. Three cases were treated with antimicrobials (all three had azithromycin and one also had tetracycline). All four recovered from cholera. The holy water consumed in the UK was disposed after onset of symptoms. There was one symptomatic co-traveller who was not tested for cholera as they recovered without medical intervention.

## Phylogenetic analysis and genome-derived antimicrobial resistance determinants using whole genome sequencing

**Figure 1:**
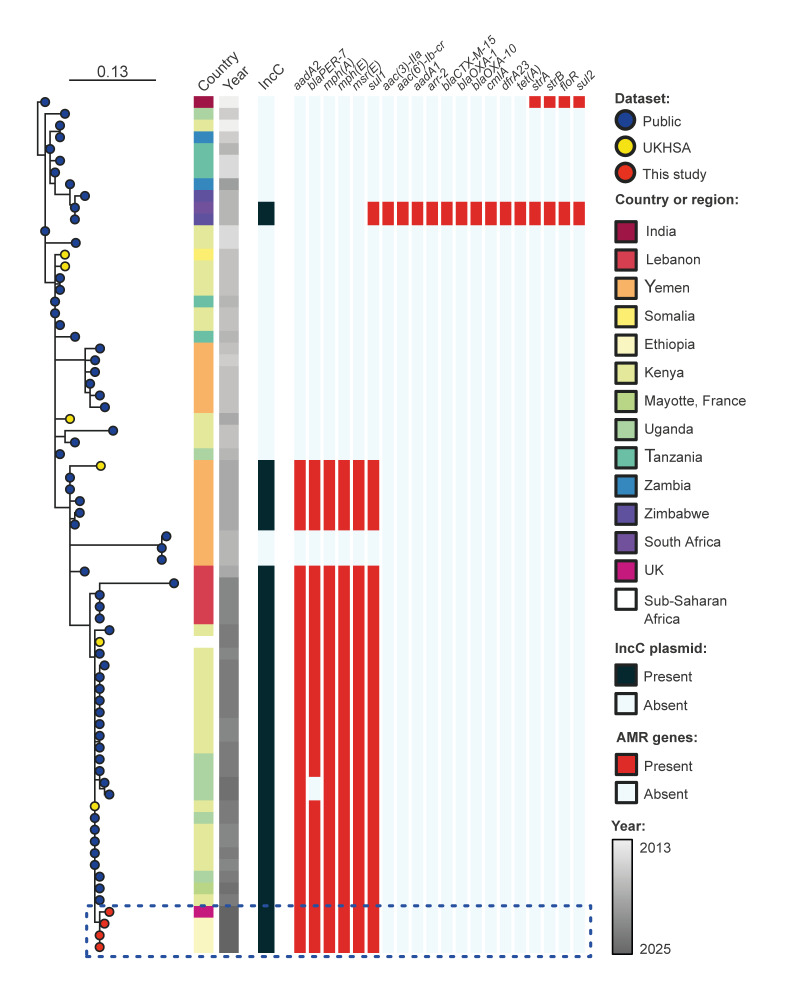
Maximum-likelihood phylogenetic tree of *Vibrio cholerae* O1 7PET AFR13 sub-lineage generated from a core genome single nucleotide polymorphism (SNP) alignment, United Kingdom (n = 4) and other countries (n = 69), 2014–2025^a^

AMR: antimicrobial resistance; UK: the United Kingdom; UKHSA: UK Health Security Agency.

^a^ Isolates from the German patients were not included in the analysis.

Phylogenetic analysis using core genome single nucleotide polymorphism (SNP) showed all four enteric isolates from the UK residents belonged to a previously described MDR clade of *V. cholerae* O1 comprising isolates linked to outbreaks of cholera in Kenya and Sub-Saharan Africa, as well as Eastern and Middle Africa (Figure 1) [4].

The four isolates sequenced in the United Kingdom associated with Ethiopia clustered together (in blue box) within a larger clade containing other genomes from Eastern Africa, alongside corresponding metadata and antimicrobial resistance gene presence.

The Ethiopian *V. cholerae* isolates sequenced by RKI and UKHSA had the same genotypic antimicrobial resistance (AMR) profile as the previously reported Kenyan and Sub-Saharan isolates from UK residents [5]; *gyrA*S83I, *parC*S85L, *dfrA1*, *catB9*, *aadA2*, *bla*_PER-7_, *mph(A)*, *mph(E)*, *msr(E)*, *sul1*, *qacEΔ1*, likely to confer resistance to fluroquinolones, trimethoprim, chloramphenicol, aminoglycosides, beta-lactams, macrolides and sulphonamides. This strain also carried the same *IncC* conjugative plasmid with the Yem*VcH*MDRI resistance island encoding genes responsible for resistance to streptomycin and spectinomycin, cephalosporins, macrolides and sulphonamides [4-7]. The genetic relatedness and identical AMR profiles for the Ethiopian isolates, as well as the previous Kenyan and Sub-Saharan African strains isolated in England, provided evidence of transmission into Europe of the MDR *V. cholerae* O1 clones circulating in Africa (Figure 1).

## Discussion

Cholera is a gastrointestinal infectious disease caused by *V. cholerae* O1 or O139 carrying the cholera toxin gene. In the European Union (EU), in the 10 years before the COVID-19 pandemic and pre-Brexit (2010–2019), 9–35 confirmed cases of cholera per year were notified (median: 22 cases). In these 10 years, most cases were notified in the UK (n = 145), followed by Germany (n = 18) and France (n = 16) [4]. After the pandemic was officially declared over in May 2023, the WHO reported a global upsurge in cholera cases, an increase in the number of countries reporting cases, including reports of large outbreaks and ECDC reported a 10-fold increase in the number of imported cholera cases in Europe in 2022 compared with 2021 [8,9]. Almost all cases in Europe are travel-associated, but autochthonous cases due to consumption of often privately imported foods and water from cholera areas have been reported. Prior to the current cluster, the last such cases to occur in the EU were: in 2022 after consumption of food imported from Cameroon, and in 2001, a patient was infected in Germany following consumption of privately imported fish from Nigeria [8,10].

Cholera can be fatal if left untreated. The mainstay therapy is oral rehydration; however, antimicrobials maybe be recommended to reduce the severity and duration of the illness. Treatment options include tetracycline (the drug of choice for the treatment of cholera), fluoroquinolones and macrolides [10,11]. The *V. cholerae* O1 isolates described in this report were MDR but remained susceptible to tetracycline. Nevertheless, there is documented evidence that another AFR13 strain harbouring a similar *IncC* plasmid previously acquired *tetA* conferring resistance to tetracycline in Zimbabwe [12]. There are increasing reports of isolates from MDR AFR13 clades being imported into Europe by travellers returning from endemic regions [4].

As the infectious dose of *V. cholerae* O1 has been estimated to be 10^5^–10^8^ colony-forming units (CFU), this suggests the holy water was heavily contaminated and bacteria remained viable at ambient temperature during the flight and in Europe. Holy water consumption has been identified as a risk factor for cholera in Ethiopia previously, and preventive measures, including training, risk communication and community engagement have been taken around religious holidays involving access to holy water in Ethiopia as recent as 2024 [13]. The Ethiopian National Guideline for Cholera Surveillance and Outbreak response also address the cholera-risks associated with water from holy wells [14-16].

Travellers eating food cultivated in areas where cholera is endemic should follow the ‘cook it, peel it or forget it’ rule, ensure drinking water is bottled or boiled, and refrain from transporting food and/or water across borders. In the case of holy water, purely external use would reduce gastrointestinal infection risks. Regarding the current cholera outbreak in Ethiopia and its reach to Europe, since all cases described identified as having Ethiopian ethnicity, cholera should currently be considered in the differential diagnosis and consumption of holy water queried in patients with profuse watery diarrhoea and a similar background but no travel history.

## Conclusion

The extension of a cholera outbreak in Africa causing a cluster of infections in Europe is unusual. Genomic data have improved surveillance, case finding, identification of outbreaks and monitoring of global transmission and AMR. However, the prevention of cholera requires sustained investment in water, sanitation and hygiene (WASH). Low-income countries will continue to need overseas development aid support to control outbreaks and epidemics using effective WASH, surveillance, communications, diagnostics and countermeasure programmatic delivery.
